# Weekly Doxorubicin Increases Coronary Arteriolar Wall and Adventitial Thickness

**DOI:** 10.1371/journal.pone.0057554

**Published:** 2013-02-21

**Authors:** Delrae M. Eckman, R. Brandon Stacey, Robert Rowe, Ralph D′Agostino, Nancy D. Kock, David C. Sane, Frank M. Torti, Joseph Yeboah, Susan Workman, Kimberly S. Lane, W. Gregory Hundley

**Affiliations:** 1 Department of Pediatrics, Wake Forest University School of Medicine, Winston-Salem, North Carolina, United States of America; 2 Department of Internal Medicine (Section on Cardiology), Wake Forest University School of Medicine, Winston-Salem, North Carolina, United States of America; 3 Department of Biostatistical Sciences, Wake Forest University School of Medicine, Winston-Salem, North Carolina, United States of America; 4 Department of Pathology, Wake Forest University School of Medicine, Winston-Salem, North Carolina, United States of America; 5 Department of Internal Medicine Division of Cardiology, Virginia Tech Carilion School of Medicine, Roanoke, Virginia, United States of America; 6 Department of Cancer Biology, Wake Forest University School of Medicine, Winston-Salem, North Carolina, United States of America; 7 Departments of Internal Medicine (Section on Cardiology) and Radiology, Wake Forest University School of Medicine, Winston-Salem, North Carolina, United States of America; Virginia Commonwealth University Medical Center, Virginia, United States of America

## Abstract

**Background:**

Doxorubicin (DOX) is associated with premature cardiovascular events including myocardial infarction. This study was performed to determine if the weekly administration of DOX influenced coronary arteriolar medial and/or adventitial wall thickening.

**Methods:**

Thirty-two male Sprague-Dawley rats aged 25.1± 2.4 weeks were randomly divided into three groups and received weekly intraperitoneal injections of normal saline (saline, n = 7), or low (1.5 mg/kg to 1.75 mg/kg, n = 14) or high (2.5 mg/kg, n = 11) doses of DOX. The animals were treated for 2–12 weeks, and euthanized at pre-specified intervals (2, 4, 7, or 10+ weeks) to obtain histopathologic assessments of coronary arteriolar lumen diameter, medial wall thickness, adventitial wall thickness, and total wall thickness (medial thickness + adventitial thickness).

**Results:**

Lumen diameter was similar across all groups (saline: 315±34 µm, low DOX: 286±24 µm, high DOX: 242±27 µm; *p* = 0.22). In comparison to animals receiving weekly saline, animals receiving weekly injections of 2.5 mg/kg of DOX experienced an increase in medial (23±2µm vs. 13±3µm; *p* = 0.005), and total wall thickness (51±4µm vs. 36±5µm; *p* = 0.022), respectively. These increases, as well as adventitial thickening became more prominent after normalizing for lumen diameter (*p*<0.05 to *p*<0.001) and after adjusting for age, weight, and total cumulative DOX dose (*p* = 0.02 to *p* = 0.01). Animals receiving low dose DOX trended toward increases in adventitial and total wall thickness after normalization to lumen diameter and accounting for age, weight, and total cumulative DOX dose (*p* = 0.06 and 0.09, respectively).

**Conclusion:**

In conclusion, these data demonstrate that weekly treatment of rats with higher doses of DOX increases coronary arteriolar medial, adventitial, and total wall thickness. Future studies are warranted to determine if DOX related coronary arteriolar effects are reversible or preventable, exacerbate the known cardiomyopathic effects of DOX, influence altered resting or stress-induced myocardial perfusion, or contribute to the occurrence of myocardial infarction.

## Introduction

Cardiovascular (CV) events including myocardial infarction (MI), stroke, and congestive heart failure (CHF) are the second leading cause of premature morbidity and mortality for those surviving beyond 5 years of their initial diagnosis for breast cancer or a hematologic malignancy such as Hodgkin's disease or Non-Hodgkin's lymphoma [1]–[3]. A commonality among treatment for these cancers is the administration of anthracycline chemotherapy with agents such as doxorubicin (DOX). Several studies have identified mechanisms by which the administration of DOX is associated with myocellular injury and consequent CHF [4]–[8]. Importantly, however, there are relatively few studies that have focused on understanding potential mechanisms by which patients exposed to DOX or other anthracyclines might experience altered myocardial blood flow or sustain a MI.

To this end, we investigated the association between the administration of DOX and histopathologic changes in coronary arteriolar microcirculatory arterial segments. To address this issue, we performed a series of studies to determine the relationship between DOX administration and measures of coronary arteriolar wall thickening. In this study, we evaluated 2 doses of DOX in a rat model without coronary arterial atherosclerosis. These doses were selected to mimic doses commonly administered to patients receiving DOX for treatment of breast cancer or lymphoma. We measured the thickening of the media as well as the adventitia of the coronary microcirculatory arteriolar segments.

## Methods

### Study Design

This study was performed at the Wake Forest University School of Medicine as part of a protocol approved by the Institutional Animal Care and Use Committee (Assurance #A04-137) of Wake Forest University, and was funded by the National Institutes of Health (study identifier R21CA109224). A total of thirty-two male Sprague-Dawley rats (a subset of animals previously reported [9]), aged 25.1±2.4 weeks, were randomly assigned into one of three groups: Group 1 (n = 7) served as our control group and received weekly intraperitoneal injections of normal saline (saline); Group 2 (n = 14) received weekly intraperitoneal injections of a low dose of DOX (low DOX, 1.5 mg/kg/week, n = 12 or 1.75 mg/kg/wk, n = 2); Group 3 (n = 11) received weekly intraperitoneal injections of a high dose of DOX (2.5 mg/kg/week).

At the 2, 4, 7, and 10–12 week intervals, rats from the DOX treatment groups were randomly selected for necropsy and histopathologic examination of the coronary arteriolar and adventitial diameters. Histopathological examination of saline treated animals was conducted at 2 weeks in 1 animal and at the end of the 10 week study in the other 6 animals. During receipt of saline and DOX, rat physical appearance and weights were routinely monitored. If the physical appearance of rats reached a level of 5 or body weight decreased greater than 25% from the start of study (see Table 1), animals were considered to be in poor condition (near death), withdrawn from the study and euthanized.

**Table 1:** Effect of DOX on rat physical parameters and EF measurements (mean ± standard error of the mean.

Treatment	Weeks in study	Rat weight, gm	Physical Appearance score	EF Measurements
**Saline**	0	483±37 (n = 7)	1.0±0.0 (n = 7)	77±3 (n = 7)
	2	488±35 (n = 7)	1.3±0.3 (n = 7)	73±2 (n = 5)
	4	521±30 (n = 6)	1.0±0.0 (n = 6)	77±3 (n = 6)
	7	538±29 (n = 6)*	1.0±0.0 (n = 6)	78±2 (n = 5)
	10+	544±29 (n = 6)*	1.0±0.0 (n = 6)	77±1 (n = 4)
**Low Doxorubicin (1.5–1.75 mg/kg/wk)**	0	415±12 (n = 14)	1.0±0.0 (n = 14)	77±1 (n = 14)
	2	411±12 (n = 14)	1.9±0.1 (n = 14)	75±1 (n = 12)
	4	401±14 (n = 9)	2.0±0.0 (n = 9)	73±3 (n = 6)
	7	389±13 (n = 8)**	3.8±0.4 (n = 8)#	73±3 (n = 8)†
	10+	368±16 (n = 3)**	3.3±0.3 (n = 3)#	67±9 (n = 3)†
**High Doxorubicin (2.5 mg/kg/wk)**	0	467±17 (n = 11)	1.0±0.0 (n = 11)	70±2 (n = 11)
	2	460±15 (n = 11)	1.8±0.1 (n = 11)	69±2 (n = 9)
	4	418±11 (n = 11)	3.4±0.2 (n = 11)#	65±3 (n = 11)
	7	408±10 (n = 5)**	4.4±0.4 (n = 5)#	55±6 (n = 4)‡
	10+	438±0 (n = 1)	3.0±0.0 (n = 1)#	74±0 (n = 1)

Number of animals in treatment group at any given time is indicated within the parentheses.

Average appearance score of animals in study, where 1  =  excellent, active; 2  =  good: active, slight hair loss; 3  =  fair: less active, slight bloating; 4  =  poor: reluctant to move, poor appetite and diarrhea; 5  =  critical: marked pallor, lethargic.

* increase in weight relative to baseline *p*<0.0001

** decrease in weight relative to baseline *p*≤0.001

#increase in appearance score relative to baseline *p*<0.0001

† decrease in LVEF relative to baseline *p* = 0.005

‡ decrease in LVEF relative to baseline *p* = 0.01

### Animal Euthanasia and Pathology Data

**Figure 1:**
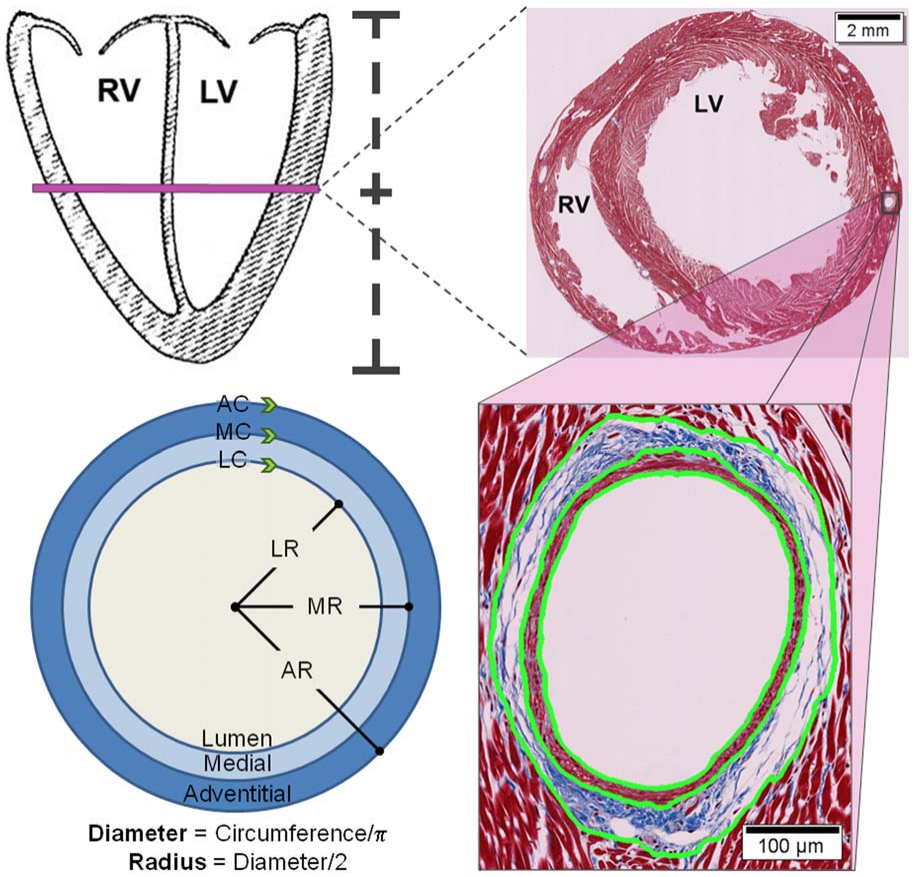
Acquisition of histological samples. Schematic representation that illustrates the location, selection, and analysis method of the histological specimens used to measure arteriolar wall thickness. Sectioned histologic specimens were obtained in a short axis plane located ½ the distance between the mitral annulus and the apex of the left ventricle (top left). In the top right panel, a low resolution image of the rat myocardium at 2x with high resolution is shown with an insert of a coronary arteriolar vessel at 20x (bottom right). Values for individual artery lumen, medial and adventitial circumferences (LC, MC, and AC, respectively) were obtained by tracing the region of interest using the digital imaging analysis program NDP.view (indicated in green, bottom right). The bottom left panel shows how the circumference data were used to find the lumen, medial, and adventitial radii (LR, MR, and AR, respectively) in order to calculate average diameters and wall thickness.

After animal euthanasia (ketamine/xylazine 80/12 mg/kg IM followed by CO_2_ asphyxiation), gross examination of the rats was performed. The hearts were excised, the aorta was cannulated, and then the heart was mounted on a Langendorff apparatus, pressurized to 52 cm H_2_O, and perfused with paraformaldehyde for 20 minutes. The heart was then transferred to chilled paraformaldehyde for 24 hours followed by a solution of 70% ethanol. Appropriate sections were trimmed to obtain the middle short axis view. As shown in Figure 1 (upper left panel), this section was obtained at ½ the distance from the mitral annulus to the cardiac apex. The sections were embedded in paraffin, processed routinely for histology, cut at 4 to 6 µm, and stained with Mason's trichrome to clearly visualize the smooth muscle and adventitial boundaries (Figure 1, upper right and lower right). Images were digitally scanned utilizing a Hammatsu Nanozoomer 2.0 HT virtual imaging system equipped with an Olympus objective x40 NA 0.75 (Digital Imaging Core, Wake Forest University School of Medicine) and resolved at 40X to 60X utilizing Nanozoomer Digital Pathology Virtual Slide Viewer (NPD.view, version 1.2.36, Hammatsu Photonics KK, Japan) to assess lumen, medial and adventitial circumferences of the 3 largest, similar caliber intramyocardial arteries. Values for individual artery lumen, medial and adventitial circumferences (LC, MC, and AC; respectively) were obtained by tracing the region of interest (NDP.view) as indicated by the green arrowheads in Figure 1 (lower left panel). Values for LC, MC, and AC were used to obtain specific diameters, radius and wall thickness values as indicated below:

Lumen calculations:

- Average Lumen Diameter (LD)  =  LC/π
- Average Lumen Radius (LR)  =  LD/2

Medial calculations:

- Average Medial Diameter (MD)  =  MC/π
- Average Medial Radius (MR)  =  MD/2
- Average Medial Thickness (MT)  =  MR - LR

Adventitial calculations:

- Average Adventitial Diameter (AD)  =  AC/π
- Average Adventitial Radius (AR)  =  AD/2
- Average Adventitial Wall Thickness (AT)  =  AR – MR

Full Thickness (FT):

- FT  =  MT+AT

Lumen diameter (LD), medial wall thickness, adventitial thickness, and full thickness (medial thickness + adventitial thickness) were calculated and presented in the absence (Table 2) and presence (Table 3) of normalization to LD.

**Table 2:** Effect of Doxorubicin (DOX) on coronary artery lumen diameter, medial thickness, adventitial thickness and total wall thickness.

Treatment	Number of animals in analysis	Lumen Diameter (µm)	Medial Thickness (µm)	Adventitial Thickness (µm)	Total Wall Thickness (µm)
**Saline**	7	315±34	13±3	23±4	36±5
**Low Doxorubicin**	14	286±2	12±2	24±3	36±4
**High Doxorubicin**	11	242±27	22±2**	28±3	51±4*

Low Doxorubicin: 1.5 mg/kg to 1.75 mg/kg

High Doxorubicin: 2.5 mg/kg

Values are expressed as the unadjusted mean ± SEM. All statistical comparisons shown are between different doxorubicin doses to normal saline (*p = 0.02, **p = 0.005 in comparison to normal saline).

**Table 3:** Effect of Doxorubicin (DOX) on coronary artery medial thickness, adventitial thickness and total wall thickness: normalized to lumen diameter.

Treatment	Number of animals in analysis	Medial Thickness/LD	Adventitial Thickness/LD	Total Wall Thickness/LD
**Saline**	7	0.04±0.01	0.07±0.02	0.12±0.03
**Low Doxorubicin**	14	0.04±0.01	0.09±0.02	0.14±0.02
**High Doxorubicin**	11	0.10±0.01***	0.14±0.02 *	0.25±0.02**

Low Doxorubicin: 1.5 mg/kg to 1.75 mg/kg

High Doxorubicin: 2.5 mg/kg

Values are expressed as the unadjusted mean ± SEM. All statistical comparisons shown are between different doxorubicin doses to normal saline (*p<0.05, **p<0.01, and ***p<0.001).

### Statistical Analyses

Animals within each group were assessed at pre-specified time intervals. All analyses that compared groups at a fixed point in time were performed first using analysis of variance (ANOVA), then using analysis of covariance (ANCOVA) to adjust for age, weight, and the total cumulative dose of DOX. In addition, separate analyses were performed to account for the potential influence of time or duration of treatment on our results. Only when there were overall significant differences in appearance among the 3 groups would pair-wise comparisons of least means squares be tested with Student's t-tests between the saline and the low- and high-DOX groups. Comparisons were made to determine whether the thickness of the adventitia, thickness of the media, and the total thickness of the media-adventitia differed between DOX and saline treatment groups. All values are reported as mean ± the standard error of the mean (SEM), and unless stated otherwise, a value of *p*<0.05 was considered significant. For all analyses, SAS version 9.1 was used.

## Results

Characteristics pertaining to the animals are provided in Table 1. As shown, the animals receiving saline remained healthy throughout the study. Those receiving low DOX developed signs of poor health at 2 weeks that became more severe by 7 weeks into the study (*p*<0.0001; Table 1). Those animals receiving 2.5 mg/kg/week also experienced signs of poor health that began 2 weeks into receipt of DOX and worsened by the 4^th^ week of study (*p*<0.0001; Table 1). In animals receiving either low or high weekly doses of DOX, LVEF declined relative to baseline (*p* = 0.01 to 0.005; Table 1).

**Figure 2:**
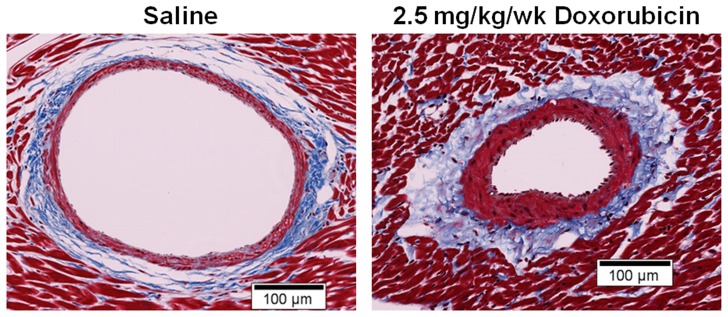
Coronary arteriolar histology in saline versus Doxorubicin sample. Coronary arteriolar histology from an animal treated with normal saline (1cc/week) for 7–10 weeks (left) with a coronary arteriole obtained from an animal treated with 2.5 mg/kg/week of doxorubicin for 10 weeks (right). Compared with the normal saline treated artery, the doxorubicin treated artery has increased medial and adventitial thickness.

Lumen diameter, medial wall thickness, adventitial thickness and total arterial wall thickness are shown in Table 2. Values for LD in those animals receiving saline (n = 7), low DOX (n = 14) and high DOX (n = 11) were similar (*p* = 0.22, Table 2). As shown, animals exposed to high DOX exhibited an increased medial thickness (*p* = 0.005), and total thickness (*p* = 0.02) compared to animals receiving saline (Figure 2 and Table 2).

**Figure 3:**
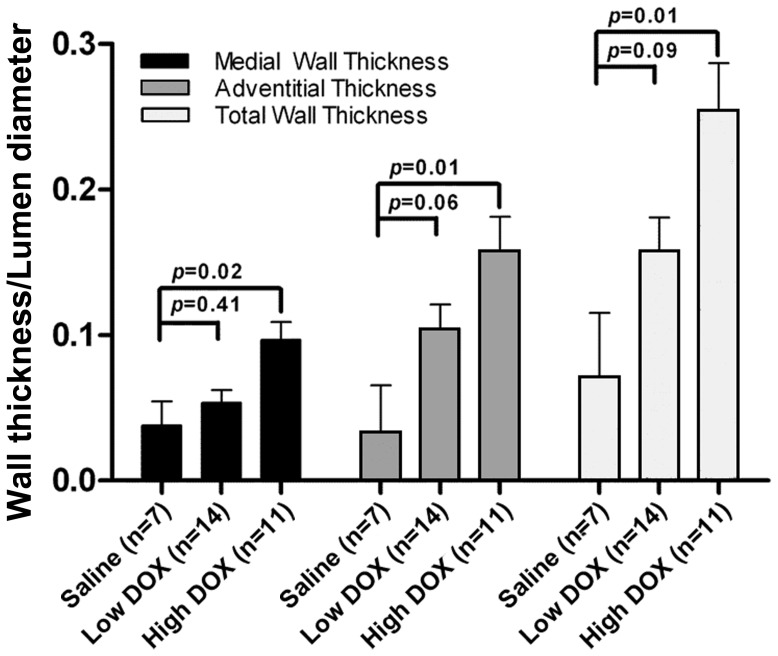
Adjusted medial, adventitial, and total wall thickness. These data summarize the effect of saline (n = 7), low (1.5 mg/kg to 1.75 mg/kg, n = 14) or high (2.5 mg/kg, n = 11) doses of doxorubicin (DOX) on coronary arteriolar medial, adventitial, and total wall thickness after normalization to lumen diameter and adjusting for animal age, weight, and duration of treatment. Data shown are adjusted mean ± the standard error of the mean (SEM). P-values are provided for comparisons between saline and doxorubicin treated groups (analysis of covariance).

When normalized to LD (Table 3), high DOX animals experienced an increase in medial wall thickness, adventitial thickness and total wall thickness (*p*<0.05 to <0.001, Table 3). We assessed the medial, adventitial, and total wall thickness normalized to LD after accounting for potential differences in age, amount of DOX received and treatment duration by the animals. As shown in Figure 3, there were trends toward an increase in adventitial and total wall thickness in the animals receiving low DOX. In animals receiving high DOX, medial, adventitial, and total wall thickness were elevated relative to saline controls after accounting for age, weight and total dose of DOX received (Figure 3).

To examine the potential influence of treatment duration on wall thickness values, we categorized duration of exposure into 3 intervals (2 or 4, 7 and 10 weeks). Because of the relatively few numbers of animals available for study of the 2 week interval, the animals within the 2 and 4 week groups were combined. For the high DOX animals, those with the shortest duration of exposure (2 or 4 weeks) had the highest levels of adventitial, medial and total wall thickness relative to those treated for 7 or 10 weeks. This observation was strongest for the adventitial measures (*p*< 0.009 for comparisons) followed by the total wall thickness (*p*<0.04 for comparisons). In the low DOX and saline groups, the effect of duration of treatment was not significant for any thickness measure throughout the duration of the study.

## Discussion

The results of this study indicate: a) the administration of 2.5 mg/kg/week of DOX increases the thickness of the walls of coronary arterioles of Sprague-Dawley rats, driven primarily by an increase in medial thickening (Table 2); b) these increases persist after indexing the regions of wall thickness for the LD of the vessel, and after accounting for animal age, weight, and the total cumulative amount of DOX received (Table 3 and Figure 3); c) when duration of treatment is assessed, those animals receiving 2.5 mg/kg/week for ≤4 weeks experience greater amounts of arterial thickening than those surviving for ≥10 weeks of treatment; and d) after adjustment for age and DOX dose, lower doses of DOX (1.5 mg/kg/week) trend toward an increase in the total wall thickness of coronary arterial segments (Figure 3). This trend is primarily related to trends in an increase in the thickening of the adventitia (Figure 3).

Anthracycline-based chemotherapeutic regimens (including those that incorporate DOX) are among the most widely used regimens to treat malignancies. Today, more than 60,000 patients receive anthracyclines each year to treat leukemia and lymphoma, and breast, uterine, ovarian and lung cancer [10]–[12]. One-half of all patients exposed to anthracyclines, including DOX, demonstrate some degree of cardiac dysfunction 10 to 20 years after chemotherapy, and 5% of them develop overt CHF [11]. To date, much of the research into the primary mechanism of anthracycline cardiomyopathy has focused on the direct effect of anthracyclines on the left ventricular myocytes [5], [13], [14], with prior histologic studies (including one from our group) demonstrating evidence of disorganized myofibrillar morphology, myocellular vacuolization and necrosis, and interstitial fibrosis [9], [15].

In addition to CHF, however, anthracycline exposure has been found to increase the incidence of vascular events including MI and stroke [1], [2], [16]. This issue has become highly relevant as the number of cancer survivors has increased significantly over the past 20 years [17], [18]. For example, among 10-year survivors of breast cancer, data from 1970 through 1986 indicate that women having received adjuvant anthracycline-based chemotherapy experience an increase in MI (hazard ratio [HR] = 2.55, 95% confidence interval [CI] = 1.55–4.19; *p*<0.001), and stroke (HR = 1.85, 95% CI = 1.25–2.73; *p* = 0.002) relative to women without cancer or the prior receipt of an anthracycline [11]. As recently recognized by the National Cancer Institute, important trends are emerging regarding cancer treatment: as advanced therapeutics are reducing cancer-related illness, some of this benefit is offset by an adverse rise in morbidity and mortality from CV disease [3]. This paper presents new data that describes the effect of DOX exposure on the thickening of the walls of the coronary arterioles in hopes of determining whether acute exposure to DOX influences the coronary arteriolar microcirculation.

As demonstrated in Table 1, animals in this study developed an unhealthy appearance, a decrease in weight, and a drop in LVEF upon receipt of DOX. These findings are similar to those observed in prior studies [19]. As shown in Table 2, total microcirculatory arteriolar wall thickness increased after the 2.5 mg/kg/week dose of DOX. This increase in arterial wall thickness was accounted for primarily by an increase in the medial component of the wall (Figure 2 and Table 2), and remained present after indexing the size of the arteriolar segments for the diameter of the vessel lumen (Table 3). The results after indexing indicate that the increases in wall thickness were not confined to smaller or larger arterioles and not related to potential differences in vessel sizes between our treatment groups (saline, low and high dose DOX).

Since the animals in the study exhibited differences in age, weight, and total cumulative DOX dose, we also performed analyses to account for these differences. As shown in Figure 3, increases in wall thickness after high DOX exposure persisted after accounting for the age and weight of the animals as well as the cumulative dose of DOX received. Although not statistically significant, strong trends were respectively observed (*p*<0.06 to 0.09) in increases in adventitial and total wall thickness in animals receiving lower doses of DOX after accounting for age, weight and cumulative DOX dose.

There are several mechanisms which may explain the coronary arteriolar wall thickening observed in this study after DOX. Adventitial hypertrophy has been linked with oxidative inflammation, most notably NADPH oxidase [20]. Anthracyclines are known to increase free radical production via effects on NADPH oxidase [21]. Adventitial inflammation with increased NADPH oxidase activity causes significant adventitial fibrosis and smooth muscle hypertrophy [20]. Inflammation in the adventitial layer can exhibit strong paracrine effects on both the media and intima, and as a result, stimulate smooth muscle hypertrophy within the walls of the arterial segments [20], [22].

In addition to a primary effect of DOX on the media of the arterioles, other comorbidities may be exacerbated by DOX that indirectly promote increased arteriolar wall thickening. For example, increased medial thickness may result chronically from pressure overload such as elevated blood pressure which may result from increased arterial stiffness [23]. In this study, we did not measure blood pressure, nor did we routinely assess renal function; thus we do not have data that can address this issue.

The results of this study do not provide mechanisms to account for the increased risk of subsequent MI after anthracycline exposure. Other study results, however, have demonstrated associations between anthracycline exposure and vascular dysfunction in non-coronary vascular beds [24]–[28]. Bar-Joseph, et al, demonstrated vascular dysfunction acutley after exposure to DOX, and Murata, et al, demonstrated effects of DOX on cultured endothelial cells [27]–[28]. Inflammation from injury to the tunica media precipitates intimal hyperplasia [29], which in other studies has preceded acute coronary artery syndromes [30]. In addition, medial layers with increased thickness are not able to respond to nitric oxide to augment blood flow [31]. As such, increased medial and adventitial thickening may contribute to abnormal myocardial perfusion. Our results indicate that increased coronary artery wall thickening occurs early after exposure to the DOX, and suggests further studies are warranted to investigate the mechanism of this association and whether this increase in wall thickening is associated with reduced vasodilator reserve, premature atherosclerosis, or increased myocardial ischemia in the setting of CV stress.

There are several limitations to this study. First, this may be a phenomenon isolated to rats. As such, subsequent studies will need to be employed in other animal models and human participants to document the occurrence. Second, we are uncertain if this response would occur using other anthracycline preparations. Further studies will need to be performed to see if these changes persist with encapsulated DOX, which is used for human treatment. Third, this study focused on the anatomy of the involved coronary arterioles. Future studies need to also describe the physiologic function of coronary arteries as well as the smaller micro vessels exposed to anthracyclines.

In conclusion, early after exposure to anthracycline-based chemotherapy with DOX, microcirculatory coronary arteriolar wall segments develop increased thickening of the medial and adventitial layers leading to an overall increase in microcirculatory arteriolar wall thickness. Further studies are indicated to determine the etiology of this occurrence and whether this finding is permanent and promotes myocardial ischemia in the setting of vasodilator or exercise induced stress.
